# A cheap and novel way of decompressing obstructed large bowel

**DOI:** 10.1308/rcsann.2014.96.1.83

**Published:** 2014-01

**Authors:** M Nixon, M Kostalas, D Williams

**Affiliations:** North Devon Healthcare NHS Trust,UK

Despite the introduction of stents for large bowel obstruction, many patients still require an emergency laparotomy.^1^ The colon is often decompressed using a Savage or pool sucker. A Robinson drain provides an effective alternative. Extra side holes are cut in the drain, which is then connected to suction. It can be inserted through the terminal ileum into the caecum via the ileocaecal valve, avoiding damage to the fragile colon. In our experience, the drain blocks less frequently than other methods. It can be left in situ during surgery owing to its pliability. It is also readily available and cheap.

**Figure 1:**
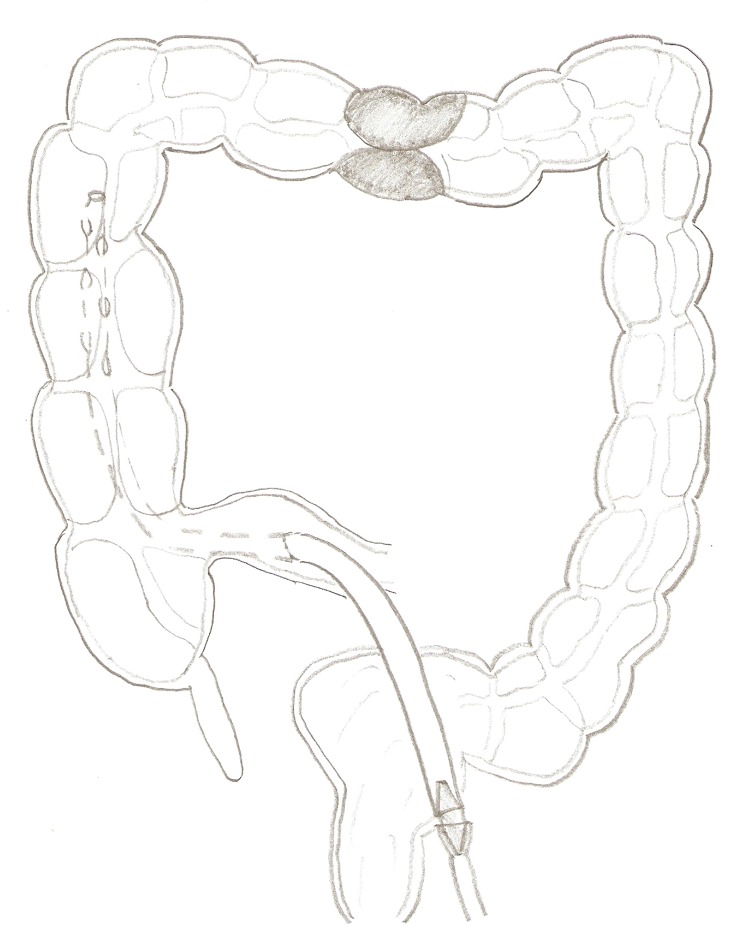
Robinson drain in situ
